# To Boost or Not to Boost: Acceptability of a COVID-19 Booster Dose among Osteopathic Medical Students: A Cross-Sectional Study from a Medical School in New York

**DOI:** 10.3390/epidemiologia3020017

**Published:** 2022-04-26

**Authors:** Taysir Al Janabi, Maria Pino

**Affiliations:** New York Institute of Technology, College of Osteopathic Medicine (NYITCOM), Glen Head, NY 11545, USA; mpino@nyit.edu

**Keywords:** COVID-19, COVID-19 vaccine, COVID-19 booster, osteopathic medical students, medical students, vaccination, pandemic, vaccine uptake, healthcare workers

## Abstract

The COVID-19 pandemic continues to evolve, with new variants emerging and vaccine-induced immunity waning. Protecting and retaining the healthcare force remains crucial in fighting this pandemic, as healthcare workers (HCWs) are a critical driver in increasing vaccine uptake among the public. This study explored the uptake of severe acute respiratory syndrome coronavirus 2 (SARS-CoV-2) booster shots among medical students at the New York Institute of Technology College of Osteopathic Medicine (NYITCOM). Predictors for actual booster uptake were also examined. An electronic survey was distributed to Osteopathic Medical Students (OMS I-IV) in January 2022. The survey was distributed to 1762 students total, with 319 responses received (18%). Of those who responded, 70.2% (224/319) reported that they had already received a booster, while 29.5% (94/319) reported they had not yet received it. We identified that pharmaceutical mistrust, building long-lasting immunity via vaccines, and vaccines’ adverse effects were the most significant predictors for how willing participants were to accept a booster dose. Vaccine hesitancy around the COVID-19 booster was prevalent during the surge of the highly transmissible variant Omicron. This finding necessitates some evidence-based approaches to enhance booster uptake among a population subgroup whose impact is critical.

## 1. Introduction

The coronavirus disease 2019 (COVID-19) pandemic, which was caused by the severe acute respiratory syndrome coronavirus 2 (SARS-CoV-2), has inflicted significant mortality and morbidity across the globe [1,2]. With the arrival and approval of new COVID-19 vaccines, the world has seen hope in the fight against this pandemic [3]. Several public health measures have made significant contributions to the reduction in morbidity and mortality of many diseases [4], with vaccination being a fundamental contribution to the prevention and elimination of many infectious illnesses [4,5]. Moreover, vaccination offers considerable social, economic, and health benefits [6]. With the approval of highly effective vaccines—Pfizer–BioNTech and Moderna messenger RNA vaccines (mRNA)—in December 2020 by the United States (US) Food and Drug Administration (FDA), a historic vaccine campaign began that has been complex in both scope and complexity [7].

Despite the huge investment from the US government in vaccine distribution in December 2020, the initial vaccine rollout was challenging because of limited vaccine supply, delayed delivery of federal resources to the states, and other logistical reasons [8,9]. While the pace of immunization has increased steadily since the start of the COVID-19 vaccine campaign, the rate of vaccination might not have been adequate to halt the emergence of more virulent viral strains [10]. Additionally, Patel et al.’s research observed that the removal of nonpharmaceutical interventions (NPIs), specifically mask-wearing and physical distancing, during the vaccine rollout might have contributed to the increased number of COVID-19 cases, and subsequently, the number of hospitalization and deaths [11]. A similar finding was reported by Yang et al. in China [12].

The SARS-CoV-2 virus evolves continuously, resulting in new variants that challenge existing measures of fighting the pandemic [10,13]. The SARS-CoV-2 Interagency Group (SIG), established by the US Department of Health and Human Services (HHS) seeks to improve coordination between different federal agencies and is focused on the rapid classification of emerging variants and their impact on the COVID-19 countermeasures [14]. Delta and Omicron—two variants first identified in India and South Africa, respectively—were labeled by the SIG as Variants of Concern (VOC) as they share common attributes of increased transmissibility and ability to evade vaccine-induced immunity [14,15].

Research has shown that two-dose mRNA vaccines, Pfizer–BioNTech and Moderna, are highly effective at preventing COVID-19 infections, reducing symptomatic illness and viral shedding [16,17]. However, breakthrough infections occur because of the waning of the vaccine-induced immunity over time [18]. A study in Israel has estimated that the incidence of breakthrough infections among fully vaccinated individuals is 36.5 per 10,000 people [19]. According to the US Centers for Disease Control and Prevention (CDC) report, 10,262 SARS-CoV-2 vaccine breakthrough infections occurred in fully vaccinated individuals as of 30 April 2021 [20]. The New York State Department of Health reported that there were 1,167,630 breakthrough cases of COVID-19 and 39,563 hospitalizations among fully vaccinated individuals, which corresponded to 8.7% and 0.30% of fully vaccinated individuals 12 years or older as of 22 February 2022 [21].

The more virulent variant of SARS-CoV-2, Omicron, was first identified in the US on 1 December 2021, and has been spreading rapidly; 22 states reported at least one Omicron variant case by 8 December 2021 [22]. The rapid increase in cases has been attributed to the increased transmissibility of the Omicron variant and its ability to evade immunity from either vaccination or past infection [23]. The CDC COVID Data Tracker reported that Omicron cases in the US accounted for less than 1% of new COVID cases in the first week of December, but they accounted for more than 58% of new cases by 25 December 2021 and 100% of new cases by 5 February 2022 [24].

Accumulating evidence has shown that vaccine-induced immunity wanes over time. CDC data about COVID-19 among nursing home residents observed that mRNA vaccine effectiveness against this infection declined from 74.7% in the period March–May 2021 to 53.1% in June–July 2021. Another study reported that vaccine effectiveness against infections among New York residents from 3 May to 25 July 2021 decreased for all age groups generally; however, the reduction was more significant for the age group 18–49 as effectiveness went down from 91.8% to 71.6% [25]. A technical briefing from the United Kingdom (UK) Health Security Agency reported that vaccine effectiveness of mRNA in fully vaccinated individuals declined from around 65–70% to around 10% by the fifth month after the second dose, while the booster effectiveness was 65–75%, 55–65%, and 45–50% after 2–4 weeks, 5–9 weeks, and after 10 weeks of administration, respectively [26].

Multiple studies have described that the benefits of administering a booster dose of the COVID-19 vaccine reduces the incidence of cases, severe illness, and mortality across different age groups [27,28,29]. A report published by the CDC showed that the incidence and hospitalization rate from the Omicron variant were highest among unvaccinated adults and lowest among fully vaccinated adults plus a booster [30]. With the onset of Winter in the US, the COVID-19 cases surged among vaccinated and nonvaccinated individuals. Thus, the requirement for a booster dose is justified [31].

Clinical trials have indicated the effectiveness of a booster dose in enhancing immune response after receiving mRNA vaccines six months after being fully vaccinated and two months after being fully vaccinated with the J&J/Janssen vaccine [32]. Participants in these clinical trials who received Pfizer–BioNTech and J&J/Janssen vaccine boosters were protected from severe COVID-19 infection [32,33]. The emergence of the Omicron variant, travel during the holidays, and indoor activities because of cold weather emphasize the importance of vaccination with an initial series and boosters.

This study explored the perceptions and attitudes of New York Institute of Technology College of Osteopathic Medicine (NYITCOM) medical students toward the COVID-19 booster vaccine. The World Health Organization (WHO) declared that vaccine hesitancy is a key global issue. Although this does not affect a whole population, it always involves subgroups within a population. Accumulating evidence demonstrated that vaccine hesitancy toward COVID-19 vaccines exists among healthcare workers across the world [34]. Thus, exploring future physicians’ views is critical. Research has shown that vaccine uptake among the public might be enhanced if they receive strong recommendations from their health care professionals [35]. Students can also be mobilized to respond to public health emergencies [36]. Additional factors that might influence predictors of vaccine uptake include the change in the course of the pandemic, waning immunity, circulating variants, and changes in protective behaviors against COVID-19 infection.

With this background, this study offers an opportunity to assess COVID-19 vaccine booster dose acceptance among future physicians and identify facilitators and barriers to vaccine uptake as the pandemic evolves, which can be the focus of school and health officials during vaccination campaigns during this pandemic or in the future. At the time of this study, few studies were available to address COVID-19 vaccine booster hesitancy among medical students. Thus, this study also contributes to the limited knowledge of recognizing and addressing vaccine hesitancy in a subpopulation group whose impact is paramount in protecting and promoting vaccine uptake at the individual and community levels.

## 2. Materials and Methods

The study protocol was approved by The Educational Research Data Committee (ERDC) and the Institutional Review Board (IRB) of New York Institute of Technology College of Osteopathic Medicine (NYITCOM) (protocol code BHS1712 on 01/10/2022). NYITCOM has 1762 medical students enrolled in total; 51.3% of the students are female, distributed between preclinical (OMS I-II) and clinical (OMS III-IV) at two different campuses (Jonesboro, AR and Old Westbury, NY). A total of 72.1% (1270/1762) of the students are enrolled on the New York campus, and 27.9% (492/1762) on the Arkansas campus. The racial/ethnic composition of the school is 44.8% White, 36.8% Asian, 5.3% Black or African American, and 3.2% from multiple races.

The research team created an anonymous electronic survey by adapting a model of determinants developed by the Strategic Advisory Group of Experts (SAGE) on vaccine hesitancy, based on a systematic review of literature and immunization manager interviews [37]. The questions were adapted from similar studies [38,39,40,41,42,43,44] to reflect on vaccine hesitancy determinants—contextual, individual, group, and vaccine-specific—identified by Larson et al. [37]. Moreover, the survey was piloted to assess its validity. Additionally, the authors’ previous work in this area also evaluated the psychometric properties of the current study survey [45]. Although the authors designed the current questionnaire based on previous studies, this study has differentiated itself from others because it assessed the acceptance of COVID-19 boosters rather than the primary vaccines. It also evaluated different predictors in a different time frame when a more transmissible variant predominated. Additionally, the study also has a geographical location that is different from other studies. Moreover, the current study addressed vaccine uptake among osteopathic rather than allopathic medical students. The study’s contextual questions were confidence in the health system, experiencing/witnessing breakthrough infections, and trust in the pharmaceutical industry. The study also included five individual and group influences: seasonal flu shot uptake, experience of adverse effects from the primary COVID-19 vaccination, health beliefs, and perceived individual and occupational risk. Finally, one vaccine-specific influence—vaccination schedule—was included. The survey was distributed to all enrolled OMS using the school’s student listservs. Responses were collected over two weeks starting 11 January 2022, with a USD 1 incentive provided for each response and the total money donated to the NYIT food pantry, the Grizzly Cupboard. Participation was entirely voluntary, and the participants had the option to withdraw at any time without justifying their decision. The plan was to send an email reminder to the students one week later. However, the research team ceased the data collection phase of this study after school officials made an announcement that boosters would be required of all students, so the final analysis was based on one week of data collection.

The survey included seventeen questions with one additional conditional question about the time frame of getting a booster if participants reported that they had not received one. Questions that address confidence in healthcare, pharmaceutical mistrust, vaccine-built immunity, and vaccine schedule were measured on a 5-point Likert scale with the following answer options: “Strongly Disagree”, “Disagree”, “Undecided/Neutral”, “Agree” and “Strongly Agree”. Moreover, questions that addressed perceived risks were measured on a 3-point Likert scale with the following answer options: “Low risk,” “Intermediate risk” and “High risk”. One question that addressed the flu vaccine uptake had the following answer options: “Yes, regularly”, “Yes, irregularly” and “No, never”; meanwhile, the question that addressed the number of breakthrough infections observed had the following answer options: “0”, “1–5,” “6–10”, “11–15” and “More than 15”. Finally, a question about COVID-19 vaccines’ adverse effects had the option of “Yes/No”. Participants were also assessed on demographics (age, gender, race/ethnicity, class year, and campus location). All questions had the option of “Prefer not to answer”. If a respondent selected “Prefer not to answer” or did not provide an answer, they were removed pairwise from any analysis using that variable but still included for analysis with the variables they responded to. The race/ethnicity question was split into two binary variables, White or other and Asian or other, for inclusion in the multiple logistic regression. Analysis was performed with SPSS 27 (IBM Corporation, 2020) and statistical significance was set at *p* < 0.05. We used reflexive thematic analysis to assess the qualitative data for the question, “Why have you received it (booster) or not received it?” Two raters independently assessed each response using inductive coding for common trends and breaking those trends into overarching themes.

## 3. Results

The total response rate of the survey was 18%. About two-thirds (67.7%) of the participants (216/319) were from the Old Westbury campus, and 29.1% (93/319) were from the Jonesboro campus. Female respondents constituted 51.4% (164/319) of the study sample. Our study sample’s gender, racial/ethnic, and campus location characteristics were not significantly different from the general student population at NYITCOM as shown by Kolmogorov–Smirnov test (*p* = 0.54). Table 1 summarizes the characteristics of the study participants at both campuses.

To predict booster, the variable was coded as binary with 1 being “received booster vaccine” and 2 being “did not receive booster vaccine”, with only one person saying, “Prefer not to answer”, and thus being eliminated from the analysis.

In a logistic regression to predict booster, the only significant predictors were pharma mistrust, vaccine-induced immunity, and vaccines’ adverse effects, χ^2^ (3, N = 310) = 109.113, *p* < 0.001. If a person preferred not to answer one of those three questions, they were eliminated from the analysis. Those who strongly believed that pharmaceutical companies put financial profits over public health by producing the booster in a short period of time were less likely to have received the booster; those who believed the booster was necessary to build long-term immunity and/or did not know someone who had had a serious adverse reaction that required hospitalization (anaphylaxis, myocarditis, thrombosis) to a COVID-19 vaccine were more likely to have received the booster. This is shown in the logistic regression model in Table 2. The model produced a Cox and Snell R^2^ of 0.297 and Nagelkerke R^2^ of 0.422. With receiving the booster being the affirmative, the model had sensitivity of 92.2% and specificity of 58.7% for an overall correct prediction 82.3% of the time.

Our thematic analysis revealed common thought patterns amongst the participants (Table 3). Two groups were identified: one group, “Yes” for those who got the booster, and one group, “No” for those who did not. Of those that participated in the survey, 251 participants (78.68%) chose to answer this question, with 166 people who received the booster responding (74.10%) and 85 people who did not receive the booster responding (90.43%). A two-sample z test for proportions found that this response rate is significantly different between the two groups, with those who did not receive the booster being more likely to respond, Z = −3.2562, *p* = 0.001.

Common themes in the responses for both those who did or did not receive the booster were around booster effectiveness and immunity, along with convenience and mandate potential to a lesser extent. Within the booster effectiveness responses from the “No” group were statements relating to believing the booster is ineffective or they did not need protection, while the “Yes” group predominantly expressed belief in the effectiveness of the booster and its ability to protect them. For immunity, “No” respondents wrote about already having high immunity or immunity to COVID-19 specifically, while the “Yes” group desired greater immunity or believed their immunity may be currently low.

For mandate potential, both groups anticipated a potential mandate. The “Yes” group cited this as a reason why they already received the booster, while the “No” group was more likely to express that they would only receive the booster after it is mandated. For convenience, those in the “No” group were more likely to say they were too busy, or the booster shot was difficult to schedule, while a handful of those in the “Yes” group mentioned it was a convenient time and place for them to get the booster. Additional themes emerged in the “No” group specifically around risk and vaccine-specific concerns. For risk, this group was more likely to believe that the risk of infection or serious health risks after infection were low. For vaccine-specific concerns, there were concerns about significant side effects and whether this booster formulation is effective against common current variants. For the “Yes” group, an additional theme of moral correctness emerged, with a few participants mentioning that getting the booster was the correct thing to do or the responsible thing to do to protect others.

## 4. Discussion

The uptake of the COVID-19 vaccine booster among NYITCOM medical students who responded to the survey was 70.2% (224/319). Booster acceptance varied across different populations. The acceptance of the booster among medical students at Texas Tech University Health Science Center in Lubbock, Texas was 81.6% [46], while in Japan, the booster acceptance among medical students was 84.5% [1]. In comparison, booster acceptance among HCWs was 55.3% and 71.3% in Saudi Arabia and the Czech Republic, respectively [47,48]. Moreover, a cross-sectional study that investigated the willingness to get a booster among students in Naples, Italy, showed an acceptance rate of 85.7% [49]. This finding is comparable to the previous study, which observed that intended booster uptake was 88.9% (281/316) [50]. The difference between intended and actual booster uptake in our two studies might be explained by the time difference and the specific virus variants’ dominance. While the previous study reflected the time period of Delta variant predominance, the current work was conducted more recently when Omicron was the dominant strain. The low response rate might be explained by the early termination of the study and the lack of a reminder because of the school mandate. However, the authors believe that students with strong feelings about vaccination might have responded early to voice their motives and concerns, especially those not in favor of vaccination.

Our study found that vaccine-built immunity was a significant predictor for actual booster uptake (*p* < 0.001), a finding that is consistent with our previous work and other studies. Booster acceptance among medical students in Japan was dependent on the students’ belief in the protection and sustainability of the immunity produced by the COVID-19 vaccination series [1]. Students who believe in vaccine-induced immunity might view the booster dose as part of a process of building the optimal level of protection, so they might be more accepting of an additional COVID-19 dose than others. Medical students are more likely to be updated on the pandemic, available vaccines, and emerging variants [18,51] through their academic institutions. Thus, making them aware of reduced vaccine effectiveness and increased virus spread. Additionally, witnessing the increasing number of breakthrough infections is visible evidence of the waning of vaccine-induced immunity, especially for those in their clinical years. The rollout of COVID-19 vaccine boosters is no different from other required vaccination for medical students, which require multiple doses to achieve a high level of protection of more than 90% for both MMR (measles, mumps, and rubella) and hepatitis B [52].

Mistrust in pharmaceutical companies is a significant predictor of actual booster uptake (*p* < 0.001). This finding was consistent with our previous work [50]. Those who responded that they strongly agreed or agreed with the statement that pharmaceutical companies had prioritized financial profits over the public interest by producing the new vaccines in a short time were significantly less likely to support getting a booster shot. Medical students might view the lack of complete information about boosters’ effectiveness and the duration of its impact as a sign of lack of transparency. While a booster dose of COVID-19 enhances the immune response [29], it is still unclear how long the booster-induced immunity will last. Moreover, there is emerging evidence that boosters might lose their potency with time. The CDC reported that booster effectiveness against COVID-19 emergency/urgent care visits and hospitalization was 87% and 91%, respectively, two months after a booster dose; however, booster effectiveness declined to 66% and 78% by fourth month [53]. This decline in vaccine effectiveness might open the possibility for another booster dose in the near future.

Another finding from this study is that adverse effects from the COVID-19 vaccines significantly predicted the actual booster dose uptake (*p* = 0.003). Those who experienced or knew someone who experienced serious adverse events from the vaccine were less likely to receive the booster, a finding that was inconsistent with the literature. The number of symptoms experienced after the COVID-19 vaccination did not predict booster uptake among medical students in Israel [54]. However, our question specifically investigated the more serious adverse effects of vaccination (anaphylaxis, myocarditis, thrombosis), which might not be the main focus of other studies. University students in Italy who reported low health status after administering the primary COVID-19 series were more likely to be vaccine hesitant toward the booster dose [49]. Another study observed that medical students who had a history of anaphylaxis were less likely to get boosters [1].

It was observed that age, gender, and class did not significantly predict actual booster uptake. Our finding is consistent with the limited current literature about booster uptake among medical students [1,46]. A cross-sectional study that explored primary COVID-19 vaccine acceptance among Israeli medical students showed that gender did not predict vaccine uptake [54]. A cross-sectional study assessing the flu vaccine uptake among medical students in Saudi Arabia showed that gender and class year did not significantly predict vaccine uptake [55]. While race was not a significant predictor of booster uptake in this study, a finding contrary to our previous work, which observed that Asian race was a significant driver of booster uptake among this population [50]. A common school environment may create homogeneity of opinions, reducing the impact of demographic factors.

Perceived individual and occupational risk of contracting COVID-19 did not predict the willingness to get a booster dose (*p* = 0.989 and *p* = 0.599, respectively). The perceived risk is consistent with our previous work but inconsistent with limited literature. A cross-sectional study that addressed vaccine hesitancy toward the booster among HCWs in the Czech Republic showed that perceived risk was a predictor for booster uptake. Moreover, the mean age group of that study population was 46.9, which put them at higher risk of COVID-19 [48]. Our study sample mean age was 26.8, which put them in the low-risk category for hospitalization and death, according to a CDC report [50], which might explain the insignificance of individual risk. A high percentage of our participants shared similar occupational risk, so the lack of disparity between the respondents might have made occupational risk insignificant.

It was observed from the study that the number of breakthrough infections was not a significant predictor for the COVID-19 vaccine booster uptake (*p* = 0.926). To the authors’ best knowledge, the breakthrough infections variable has not been addressed in the current literature. However, the authors propose that this variable is not a predictor despite the increasing number of infections by the more transmissible variant, Omicron, among the vaccinated individuals because most of these infections are generally mild [56]. Moreover, vaccinated participants might still feel protected with their primary vaccine series and therefore do not perceive an urgency to obtain a booster.

There were limitations to this study. First, there was an impact to generalizability since it was conducted at one osteopathic medical school. Secondly, the school vaccine mandate limited the participant number, reducing sample size. Third, the survey was administered five days after the end of the school Winter break (6 January 2022), a time during which answering questions may not have been the students’ main priority. Lastly, information is regularly updated regarding vaccine-induced immunity and booster benefits. Thus, continuous investigation about COVID-19 spread and variants remains necessary to assess the attitudes about booster doses as perceptions can shift.

## Figures and Tables

**Table 1:** Characteristics of Study Participants (N = 319).

Variable	Number (%)
Age	
18–19	0 (0%)
20–29	260 (81.5%)
30–39	30 (9.4%)
40–49	19 (5.9%)
Prefer not to answer	5 (1.6%)
**Gender**	
Female	164 (51.4%)
Male	140 (43.8%)
Other	5 (1.6%)
Prefer not to answer	6 (1.9%)
**Race/Ethnicity**	
White	196 (61.4%)
Black or African American	9 (2.8%)
American Indian or Alaskan Native	2 (0.6%)
Asian	73 (22.8%)
Native Hawaiian or other Pacific Islander	0 (0%)
From multiple races	14 (4.4%)
Other	8 (2.5%)
Prefer not to answer	17 (5.3%)
**Class year**	
OMS I	114 (35.7%)
OMS II	93 (29.2%)
OMS III	61 (19.1%)
OMS IV	42 (13.2%)
Prefer not to answer	9 (2.8%)
**Campus location**	
Jonesboro	93 (29.1%)
Old Westbury	216 (67.7%)
Prefer not to answer	10 (3.1%)

**Table 2:** Variables in Equation.

	B	S.E.	Wald	df	*p*-Value	Exp(B)
pharma mistrust	0.574	0.156	13.475	1	<0.001	1.775
vaccine-induced immunity	−0.782	0.146	28.686	1	<0.001	0.458
vaccines adverse effects	−1.172	0.392	8.935	1	0.003	0.310
constant	2.087	1.121	3.468	1	0.063	8.062

B = Log-Odds Estimates for true beta values for each variable where log(*p*/1 − *p*) = b0 (the constant) + (b1 × 1) + (b2 × 2) + (b3 × 3); S.E = Standard Errors associated with each B value; Wald = Wald chi-square value for testing each B departs significantly from 0; Df = Degrees of Freedom; Exp(B) = Odds Ratio for the predictor Bs.

**Table 3:** Some Comments Provided by the Participants.

Theme	Representative Quotes	
	“Yes” Group	“No” Group
Booster effectiveness	“I believe that it will prevent me from becoming seriously ill.”	“I do not believe it is necessary for anyone other than the immunocompromised. Its efficacy is also questionable, just like the efficacy of the vaccines are questionable.”
Immunity	“It’s been 9 months since my last vaccine, and I assumed my immunity weaned off.”	“I already had my first 2 shots and got the Omicron variant 2 weeks ago. My understanding is that this is the best form of immunity and there is no need for further vaccines.”
Convenience	“I volunteered at a vaccination clinic and there were extra doses that I did not want to go to waste.”	“Haven’t had a convenient opportunity to get it.”
Mandate potential	“Thought it would be required later on.”	“I think it is unnecessary for my health and well-being. Furthermore, I am still unsure as to whether the booster is safe to receive. However, I will get it if NYIT requires it.”
Risk	NA	“I have not received the booster because I have been previously vaccinated against COVID-19 and I do not have any of the comorbidities that would place me at an increased risk for severe COVID-19 infection. In addition, it seems that the booster vaccine’s ability to provide substantially more protection compared to the first series of the vaccines and to prevent transmission are overstated. Similarly, with the Omicron variant as the dominant variant in NYS and its relatively mild symptoms compared to the Delta and Alpha variants, I believe that the booster vaccine would not provide me with substantially more protection that the original series of COVID-19 vaccination.”
Vaccine-specific concerns	NA	“After the second dose of the Moderna vaccine, I experienced bad side effects that resulted in the worst sickness I’ve ever experienced for a 24 h period. Even though I think boosters will be a necessary component of fighting COVID-19, I wanted to wait till the booster was updated for new virus strains before I went through the uncomfortable side effects I expect to have.”
Moral correctness	“It was the right thing to do for myself, family, and patients. There’s a lot of people in our communities who live with “invisible” comorbidities, and I wanted to do my part by protecting them as well.”	NA

## Data Availability

The data presented in this study are available upon request from the corresponding author.
